# The Bias of Physicians and Lack of Education in Patients of Color With Melanoma as Causes of Increased Mortality: A Scoping Review

**DOI:** 10.7759/cureus.31669

**Published:** 2022-11-19

**Authors:** Zehra Rizvi, Viktor Kunder, Hanna Stewart, Paola Torres, Sana Moon, Nimisha Lingappa, Mallory Kazaleh, Varshini Mallireddigari, Julian Perez, Nigel John, Anika Sedani, Robin J Jacobs

**Affiliations:** 1 Osteopathic Medicine, Nova Southeastern University Dr. Kiran C. Patel College of Osteopathic Medicine, Fort Lauderdale, USA; 2 Medical and Behavioral Research; Health Informatics; Medical Education, Nova Southeastern University, Fort Lauderdale, USA

**Keywords:** blacks, bias, health disparity, dermatology, minority patients, skin color, melanoma

## Abstract

Minorities, particularly non-White minorities, often encounter implicit biases from healthcare professionals that may impact their standard of care and quality of life. The study of dermatology has long been based on Whites, unintentionally affecting the treatment of non-White patients. Melanoma, although mostly curable, can become fatal in those presenting with advanced stages at diagnosis. Despite being rare in racial minorities, melanoma is associated with a worse prognosis among them compared to White populations. In light of this, the objective of this study was to determine the role of education in preventing biases and improving the diagnosis and treatment of melanoma in minority groups to improve patient outcomes. This study was designed as a scoping review to gather evidence on the impact of implicit bias and lack of education on the treatment of melanoma in people of color. Following Preferred Reporting Items for Systematic Reviews and Meta-Analyses (PRISMA) guidelines, we searched for peer-reviewed studies involving melanoma, education, and treatment bias in people of color on the databases PubMed, Medline EBSCO, CINAHL, and Cochrane. The data were extracted pertaining to the following main aspects: (1) risk factors, (2) surveys of current knowledge, and 3) educational interventions. This scoping review identified socioeconomic factors, bias, and lack of education in minority populations as causes of increased mortality rates in melanoma.

Moreover, because preventative dermatology is largely based on White skin types, incorporating darker skin tones into education will help dispel implicit bias. Additionally, there is evidence to indicate that current patient knowledge and understanding of skin cancer is inaccurate among many and can be significantly improved through educational interventions, such as brochures and videos. Further educational interventions may be beneficial to increase understanding of melanoma in populations of color to address health disparities in dermatological care.

## Introduction and background

In the United States, minorities, especially people of color, are often faced with implicit biases on the part of healthcare professionals that constantly affect the way their healthcare is administered, thereby affecting their quality of life [1,2]. Implicit bias refers to bias or prejudice that is subconsciously held, and it is being studied here in the context of the care of people of color, who we define as non-White racial minorities. The study of dermatology has long been based on those with White skin color, unintentionally affecting the treatment of people of color [3-5].

Even though melanoma is mostly curable, it can become fatal in those presenting with advanced stages at diagnosis [1,6]. Studies show that patients of color are two to three times more likely to die from melanoma than White patients [7]. This disparity in mortality rates between White and non-White patients stems from melanoma being identified at far more advanced stages of the disease among racial minority patients compared to their White counterparts, matched for sex and age [6]. Blacks present with deeper tumors, higher ulceration rates, and greater lymph node involvement compared to Whites [1]. Blacks are also significantly less likely to receive surgical resection treatment for localized disease, which is known to improve survival rates [1].

Colorism, the bias against darker skin tones, also plays a role in healthcare disparities related to melanoma, which is quite evident in medical education materials. Less than 5% of dermatological images in the US medical textbooks represent dark skin tones [8], and images of the common cancers associated with darker skin tones are rarely depicted [8,9]. These biases create certain beliefs about specific minority groups and non-White patients may thus be subjected to poorer quality of care based on misleading judgments regarding skin cancer based on their race. Without addressing biases and disparities in the identification, screening, and treatment of melanoma in patients of color, mortality due to melanoma in these large groups will continue to rise.

Research indicates that there are significant healthcare gaps in melanoma patients of color [2]. Currently, significant disparities exist in healthcare, starting with physician training, patient education, and access to healthcare. The lack of representation in public health education and clinical research and limited community resources have left minority communities at risk [2,10]. To address the health disparities related to melanoma among minority populations, it is important to acknowledge current implicit biases and explore the role they play in healthcare provision. This scoping review explores the available evidence about the role these biases have played in healthcare-related disparity with regard to melanoma in patients of color.

## Review

Materials and methods

A comprehensive electronic search was performed to identify studies that discuss the link between mortality in melanoma patients of color and risk factors that increase the incidence of this mortality as compared to their non-White counterparts. This scoping review utilized primary studies found via a search on the databases PubMed, Medline EBSCO, CINAHL, and Cochrane. We included studies published from 2011 to 2021 based on the inclusion and exclusion criteria described below. The selection process was performed independently by two reviewers based on the inclusion and exclusion criteria.

Search Strategy

The inclusion and exclusion criteria were established prior to performing the review. The inclusion criteria were as follows: (1) articles published during 2011-2021, (2) full texts in the English language, (3) articles involving patients aged 19 years or older, (4) studies involving minority races and ethnicities (African Americans, Hispanics, Asian Americans and Native American Pacific Islanders), (5) articles touching on socioeconomic factors or biases that impacted the outcome of mortality, and (6) articles on human subjects. Articles published before 2011, those non-specific to patients of color with melanoma, or those involving skin diseases other than melanoma were excluded. The search was conducted in December 2021 and yielded 117 results.

Identification of Studies

We used the following text words and search phrases in our search: ((melanoma) AND ((education) OR (bias)) AND ((people of color) OR (POC) OR (non-Hispanic blacks) OR (Minority) OR (African Americans) OR (Asian Americans) OR (Hispanics) OR (Skin of color) OR (Patients of color)).

Data Extraction

After screening and applying the inclusion criteria to the studies obtained from the relevant databases, all researchers organized the information on a data log that included the title, type of review and year, inclusion/exclusion criteria, sample size and age, limitations, methods, and results. The final outcomes were documented on a Google Docs spreadsheet. With the information organized, a thorough discussion of each article was conducted to determine whether it fit the inclusion criteria and fulfilled the requirements related to quality. Disagreements were resolved through discussion.

**Figure 1:**
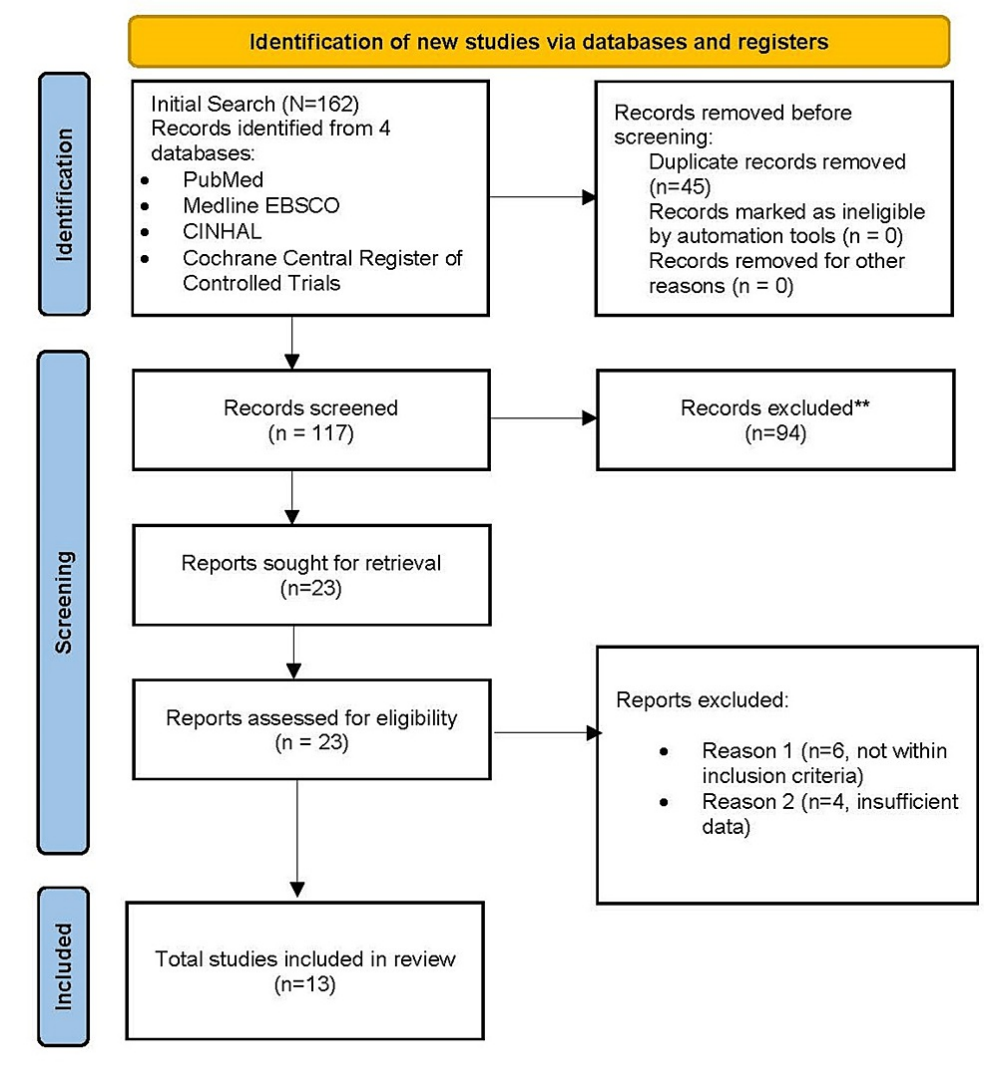
PRISMA flow diagram depicting the study selection process PRISMA: Preferred Reporting Items for Systematic Reviews and Meta-Analyses

The initial search elicited 162 articles based on the outlined search criteria. After removing 45 duplicates, an additional 94 were filtered out as they involved variables that were outside of the inclusion criteria. Once the screening process was over, the remaining articles underwent a quality assessment process, whereby 10 articles that did not match the inclusion criteria or that lacked adequate data were removed. The final articles that were selected involved socioeconomic factors and biases that influenced mortality rates related to melanoma in minorities, and included database extractions, surveys, educational intervention, randomized controlled trials (RCTs), cross-sectional surveys, and multiethnic cohort studies (Figure 1).

Results

A total of 13 studies were identified using the study selection process illustrated in Figure 1. Table 1 summarizes the characteristics of the studies included in this scoping review.

**Table 1:** The summary of findings of the selected studies AANAPI: Asian American and Native American Pacific Islander; AA: African American; SES: socioeconomic status; SSE: skin self-examination; TCE: total cutaneous examination; TSE: total skin examination

Study citation details	Study design	Purpose of the study	Measures	Key findings
Clairwood et al., 2014 [6]	Database extraction from 1992 to 2007	To analyze if the risk factor of race plays a role in delayed diagnosis of melanoma	Stages of melanoma in non-Hispanic blacks, Hispanics, and non-Hispanic whites on diagnosis	Non-Hispanic Blacks and Hispanics had a much greater incidence of later-stage melanomas than non-Hispanic Whites
Coups et al., 2013 [13]	Online survey	To analyze whether race influences how an individual conducts skin exams	Rate of conducting an SSE and getting recommended for a TCE by a physician	There is a paucity of research focusing on skin cancer prevention in the Hispanic population in the United States and this study found that most Hispanics do not use skin cancer surveillance behaviors. Only 17.6% were found to have ever performed an SSE
Dawes et al., 2016 [10]	Database extraction	To analyze how race can impact the survival rates of skin cancer	Mortality of skin cancer in White, Hispanic, AANAPI, and Black patients	The survival rates for cancer were found to be lowest to highest, respectively, for the following groups: Blacks, AANAPI, Hispanics, and White patients
Jacobsen et al., 2017 [4]	Survey - convenience sampling	To see how well minorities are educated about skin cancer and melanoma	Conceptions and misconceptions about skin cancer	An increasing minority population in the US has been associated with higher later-stage skin cancer diagnoses. This study at a free clinic in Florida involving uninsured patients living below the poverty line found several barriers to skin cancer prevention, including a belief that darker skin could not get skin cancer, not enough knowledge of skin cancer, and feeling that sun protection made them feel hot. Videos and text messages were found to be the most popular methods for public health outreach
Korta et al., 2014 [14]	Survey	To explore how race influences skin awareness and physician skin exams	Frequency of total body skin examinations; knowledge about melanoma and screening practices	Despite ethnoracial minority patients having a lower incidence of melanoma than White patients, they tend to have more advanced melanomas with decreased survival rates. In this study at a dermatology clinic in NYC, White patients were found to have had more TSEs than non-White patients. Additionally, minorities were less likely to be able to identify features that raise suspicions of melanomas. However, regardless of race, there was found to be a need for increased patient education on melanoma
Chao et al., 2017 [16]	Educational study	To Explore if education is effective in enhancing knowledge about warning signs of melanoma	Pre- and post-educational knowledge about melanoma in minority groups	The patients in the intervention group, who self-identified as AA, Asian, or Hispanic, showed a significant increase in identifying the perceived risk of melanoma, performing skin exams, and knowledge of melanoma warning signs
Mahendraraj et al., 2017 [1]	Cohort study	To provide statistics about how malignant melanoma leads to a worse prognosis in AAs	Mortality related to melanoma among different races	Despite being more common in Caucasians, melanoma has a worse prognosis in AAs. When examining differences in presentation between these patient groups, cutaneous malignant melanomas were more common in extremities in AAs and more common in the trunk in Caucasians. Five-year survival rates were lower in AAs, and deeper, advanced-stage lesions were higher in AAs
Park et al., 2012 [11]	Multiethnic cohort study	To determine whether the diagnosis of melanoma is homogenous among races	How socioeconomic background, race, and physical traits impact the diagnosis of melanoma	Risk factors typically identified for White patients such as age, sex, and phenotypic susceptibility to sunburns were evaluated in non-white populations to assess for predictive value for malignant melanoma. Excluding AAs, these risk factors were found to be predictive for non-white groups as well
Robinson et al., 2010 [3]	Focus group discussion; audiotape recording	To explore whether ethnic minorities understand the risk factors of skin cancer	Qualitative discussion about knowledge and misconceptions about skin health in ethnic minorities	The Fitzpatrick skin type classification is reliable for Whites but not well-correlated for groups such as Asians, Arabs, and AAs. Many participants did not believe they could get skin cancer and melanoma specifically was not recognized as a type of skin cancer or relevant health concern. Many minority participants were confused about the acral presentation of skin cancers among patients of color, as skin cancer is traditionally associated with sun exposure. Many participants expressed their disappointment that they did not receive adequate information about skin cancer or protection from their physicians
Roman et al., 2016 [15]	Survey and video	To evaluate the effectiveness of video educational intervention in improving knowledge about melanoma	Pre- and post-educational knowledge about melanoma in Hispanic patients	Participants scored higher on the survey after watching the educational video and some even said they incorporated the methods from the video into their daily lives. This study found that online interventions can be effective tools for education on melanoma
Sanchez et al., 2020 [5]	Cross-sectional survey	To explore how different race groups perceive melanoma	Accuracy of different race groups measuring melanoma on different Fitzpatrick skin types	Despite melanoma having a higher incidence in White patients, Hispanic patients are more at risk of an advanced diagnosis and worse prognosis. Knowledge of melanoma and its classification as a skin cancer was lowest among men, Hispanic individuals, and those with below-high school education. Only about half of Fitzpatrick skin types 3-6 could identify melanoma as cancer and even fewer knew of it as a type of skin cancer
Tsai et al., 2018 [17]	RCT (pre- and post-survey)	To see if education is effective among African American populations	Pre- and post-educational knowledge about melanoma in AAs	Sun-protective behaviors (including knowledge of perceived risk for melanoma, performing SSEs, differentiating between melanoma and other moles, and identifying asymmetry in a mole) evaluated in a cohort of AA patients showed a significant increase from pre-survey to post-survey in the video intervention group but not in the brochure intervention group. This may be due to the brochure being specifically created for darker-skinned audiences
Wich et al., 2014 [12]	Group comparison research	To analyze whether medical insurance plays a role in minority patients getting diagnosed at later stages of melanoma	Stages of melanoma diagnosis in private insurance vs. Medicaid	Melanoma patients from minority groups are known to have lower rates of survival, despite having a decreased incidence than non-Hispanic White patients. Disparities have also been linked to SES: with higher incidence linked to higher SES and worse prognosis and advanced stage at diagnosis linked with lower SES. While there is a general lack of research on melanoma among ethnic minorities, there is specifically a limited amount of information available about Asian Americans. This study found that those presenting with a lower SES and a late-stage melanoma diagnosis mostly tended to be minorities

Multiple studies showed that there is a lack of knowledge and/or misconceptions about melanoma [3-5,11-14]. People of color are more likely to believe that melanoma does not affect those with dark skin [3-4]. Studies also show that there is less understanding of self-screening practices among people of color [13-16]. 

Racial Minorities

Multiple studies in this review identified race as a risk factor in both melanoma survival and stage at diagnosis. A significantly higher proportion of advanced melanomas in non-Whites versus Whites was found, with Whites occupying the highest position in the order of survival rates, followed by racial/ethnic minority groups in the following order: Hispanics, Asian Americans and Native American Pacific Islanders, and Blacks [6,10,11].

Access to Healthcare

When looking further into this phenomenon, researchers began to focus on certain socioeconomic factors that could also be affecting the outcomes of melanoma diagnosis and survival. Healthcare coverage was identified as a risk factor among ethnic minority patients presenting to two different New York state hospitals [12]. In one hospital, 62% of patients were covered by Medicare, while at the other hospital, 72% were covered by private insurance. The patients at the primarily Medicare-covered hospital presented with a later stage at diagnosis. Since surveillance is an important factor in preventing delays in melanoma diagnosis, other studies have looked at factors that increase the likelihood of individuals completing both skin self-examination (SSEs) and total cutaneous evaluations (TCEs) by a physician. The rates found in a Hispanic cohort for SSE and TCE were 17.6% and 9.2%, respectively [13]. This study [13] also identified traits associated with individuals more likely to complete screening (as shown in the chart), which were primarily older age, English linguistic acculturation, higher levels of skincare, and higher perception of risk. A multiethnic study examining risk factors for advanced-stage melanoma diagnosis involving middle- to advanced-aged adults found that non-White/multiracial females were more likely to be diagnosed at a later stage of disease and that, overall, age and phenotypes with susceptibility to sunburn were associated with increased risk for malignant melanoma in non-White/multiracial individuals.

Educational Interventions 

Four studies in the scoping review focused on conducting surveys to assess current levels of education about melanoma among different communities [3-5,14]. A common feature of the educational surveys was a lack of understanding of the significance and likelihood of skin cancer in minority populations. Many people reported various misconceptions about melanoma, such as minorities with darker skin assuming that SSEs were unnecessary due to a decreased likelihood of developing skin cancer. Surveys also indicated problems with identifying features of melanoma but shed light on the desire to learn more about the significance of melanoma via educational videos or text messaging. 

Furthermore, three studies focused on methods of educational interventions to bridge these identified gaps [15-17]. Each of these studies demonstrated an improvement from pre-intervention to post-intervention. Some areas of improvement include understanding risk factors and prevention methods, identifying warning signs, and practicing SSEs. These results were seen across all ethnicities included in the studies. By implementing some of the methods that were discussed, awareness about melanoma can be improved throughout the community.

Discussion

The aim of this scoping review was to examine the current literature pertaining to the role biases have played in healthcare-related disparity regarding melanoma in patients of color. Following a thorough analysis and review of 13 relevant articles, we determined that there is a plethora of contributory factors that lead to the higher mortality rate in patients of color.

This can be summarized into the following aspects: (1) risk factors and patient awareness and (2) understanding of melanoma and prevention strategies. Risk factors such as socioeconomic factors, healthcare coverage/insurance, level of English proficiency, and educational status were highlighted significantly in many large database extraction studies looking at demographics and in studies looking at survey responses [11-13]. Additionally, a lack of patient awareness and understanding regarding melanoma and performing routine SSEs were reported in many of the studies investigating educational interventions [3-5,14]. When individuals were taught about melanoma (including its dangers and risk factors), they were more likely to perform SSEs, illuminating the crucial role of patient education in combating the burden of later-stage melanoma diagnosis [15-17]. These findings highlight the importance of training physicians in identifying melanoma in patients of color as their presentations typically differ from those of their White counterparts. This area was not specifically addressed in the current scoping review but can play an essential role in the underdiagnosis or later-stage diagnosis of melanoma.

The findings also highlight the need to move forward in addressing socioeconomic factors, eliminating bias, and increasing awareness and education on skin health in populations of color. Research shows that lack of healthcare coverage can be associated with increased mortality in non-White minority populations [12]. Within the scope of this study, this suggests that a significant portion of minority populations are not receiving physician-conducted skin exams, leading to later-stage melanoma diagnoses [3,14].

Medical training also plays a role in health disparities regarding melanoma and other skin cancers. Collectively, the data indicate that the preventative dermatology that exists today is inherently reflective of White skin types [3-5]. Incorporating a wide range of darker skin tones into educational materials and classification systems will help address the needs of non-White populations and help eliminate bias.

Multiple sources determined that patient knowledge and understanding of skin cancer was incomplete and inaccurate [3-5,11-14]. This is partly due to a lack of access to physicians [12], necessitating patients to look elsewhere for answers. Several studies have tested the efficacy of supplying educational brochures and videos to patient populations [15-17]. Once provided with clear and thorough instructions on how to take control of their skin health, patients have proven they can be receptive to learning and are able to integrate the information [15-17]. Further research into healthcare disparities in melanoma patients of color will help identify the causative agents of the same. Physicians and public health authorities can then work toward implementing necessary measures to decrease the mortality rate in melanoma patients of color.

Limitations of included studies

A limitation of this scoping review is the use of studies in which socioeconomic status and insurance status were not accounted for. Although these factors could have a strong correlation with the minority status of the populations in the studies, they were not included in the criteria for our review. Future research may benefit from the inclusion of these social and economic parameters. Another limitation pertains to the selection of sample populations in a few of the studies. Studies that were conducted at school health fairs and academic teaching facilities attracted more female and child participants, and those conducted at dermatology clinics attracted patients who were more receptive to melanoma education. Most of the clinic patients were college-educated and female, and hence not representative of the general population. Another limitation is the potential sampling bias. One study disregarded Hispanic populations without internet access when administering surveys and another was culturally biased in terms of communication. The study utilized terms such as burning, tanning, blistering, erythema, or freckling after sun exposure, which most participants never experienced. The language barrier created due to reliance on the English language only may have provided skewed results. Lastly, behavioral data were self-reported, raising possible concerns about recall accuracy and bias. Similarly, physicians were unable to provide information on the self-efficacy of minority patients regarding the interventions and physician education provided during their visits. This could be attributed to the study lacking a standard practice control group.

Limitations of the review process

Articles before 2011 were excluded, which removed many earlier studies relevant to this topic. Also, the application of strict inclusion and exclusion criteria may have excluded many relevant articles. Additionally, all articles that we reviewed were in the English language and addressed melanoma rates and non-White minority experiences in the United States only.

Implications for research and practice

The findings of this review point to an increased risk for non-White patients to have higher mortality rates from melanoma, despite the higher inherent risk of skin cancers in White skin. However, because higher mortality in minority patients can be attributed to non-physiological factors, there are several interventions that can potentially increase the survival rate of these patients. For example, many racial minority groups presented a lower level of understanding of melanoma as a skin cancer and of their own risk for melanoma due to the misconception that darker skin tones were not at risk for skin cancers [3,4,14].

Misconceptions about melanoma risk and detection in non-Whites can be addressed with a two-fold strategy: via education by physicians and by public health measures. Firstly, many non-White patients stated that they had never had a skin exam performed or had never been educated by a physician on the risks of skin cancer, especially in contrast to White patients’ experiences [3,13]. Educational interventions were found to be effective across several racial minority groups [15-17]. Primary care physicians can aid in promoting non-White patient health literacy by discussing sun protective measures, showing pictures of early melanoma presentations on darker skin, and starting conversations about skin cancer risks specific to darker skin tones [3]. Public health measures among minority communities should be aimed at education on melanoma with regard to its nature as a skin cancer, SSEs, visiting the physician when concerns arise, and common locations and manifestations of melanoma on darker skin [4,16,17].

In several interventions with racial minority patients, showing photographs of early presentations on different skin tones helped patients identify the risks [3,16,17]. Future research should focus on producing more information on the varying presentations of melanoma on darker skin tones and making that information a readily available resource in physician offices for both physician and patient education.

Melanomas in non-Whites versus Whites often differ in terms of common locations and symptoms. While White patients may be more informed about the association between skin cancer and increased sun exposure, patients of color will often see melanoma appear in acral locations and not correlated with sun exposure [3]. More research is needed to explore if and how sun exposure linked to melanoma in populations of color happens at similar rates as in White populations. The findings of this research could be used for public health education efforts aimed at non-White populations so that cancer prevention education and early detection are commensurate with their specific risks. Currently, many public health measures target their interventions for melanoma and skin cancers primarily in White populations [3-5,14]. Moreover, the widespread use of the Fitzpatrick skin type classification is inefficient for darker skin tones due to its language and classifications, which were set to reflect White patients' experience with sun reactivity [3-5]. More research is needed to develop skin type classifications that can better incorporate darker skin tones alongside White skin in assessments for sun reactivity and skin risk.

## Conclusions

This study used scoping review methodology to examine the literature regarding increased mortality in patients of color with melanoma. We have found evidence to suggest that socioeconomic factors, lack of access to healthcare, the presence of bias, and deficient skin cancer education among non-White populations as well as lack of physician training may contribute to the disparity in mortality rates related to melanoma in this group. These findings apply to other areas of medicine besides dermatology, where patients of color are at an increased risk for poor health outcomes. These results are concerning and warrant further research and modifications in patient and physician awareness and education.
